# 1466. Povidone-Iodine Irrigation versus Saline Irrigation for Reduction of Surgical Site Infections in Patients Undergoing Total Knee and Total Hip Arthroplasty: a large retrospective, propensity matched cohort study

**DOI:** 10.1093/ofid/ofad500.1303

**Published:** 2023-11-27

**Authors:** Samantha Simon, Andrew R Grant, Eric Smith, Brian L Hollenbeck

**Affiliations:** New England Baptist Hospital, Boston, Massachusetts; New England Baptist Hospital, Boston, Massachusetts; New England Baptist Hospital, Boston, Massachusetts; New England Baptist Hospital/ Beth Israel Lahey Health, Boston, Massachusetts

## Abstract

**Background:**

Surgical site infection (SSI) after total hip and total knee arthroplasty (THA/TKA) is a serious surgical complication. Perioperative irrigation with antiseptic compounds such as povidone-iodine have been used and are recommended by the World Health Organization and the Centers for Disease Control to reduce this risk. However, evidence supporting its superiority relative to non-antiseptic substances such as saline has been inconsistent. Therefore, we aim to identify if irrigation with povidone-iodine in THA/TKA reduces incidence of SSI in patients compared to saline irrigation.

**Methods:**

In this retrospective, cohort study of 21,482 patients who underwent TKA or THA, we compared SSI rates between patients who received povidone-iodine irrigation and saline irrigation. Univariate and multivariable analyses were performed to identify risk factors for SSI. We also performed a propensity matched analysis. Each patient’s propensity for receiving povidone-iodine irrigation was calculated using select patient and surgical variables. Standardized mean differences were calculated to confirm reduction of potential confounding between the matched groups.

**Results:**

There were 8,486 patients given saline irrigation and 12,996 patients given povidone-iodine irrigation (Table 1). In the univariate analysis, we did not identify a difference between povidone-iodine and saline (p = 0.76). Multivariate analysis showed diabetes, general anesthesia, and a procedure time of 2-3 hours increased risk of SSI, but povidone-iodine irrigation was not significantly different from saline (OR 1.1; CI 0.6-1.9) (Table 2). After propensity score matching there were 21 (0.25%) SSI in the povidone-iodine group and 19 (0.23%) in the saline group. Standardized mean differences were below 0.2 for all selected variables (Table 3). Odds of SSI were no different between the two matched groups (OR 1.1; CI 0.6-2.1).

**Table 1.**
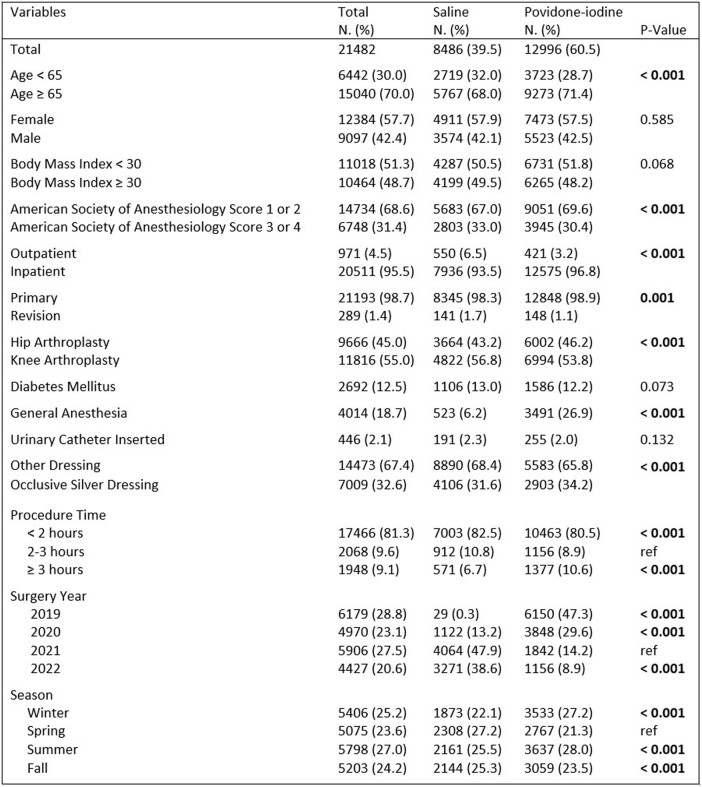
Overview of patient and surgical variables by irrigation solution.

**Table 2.**
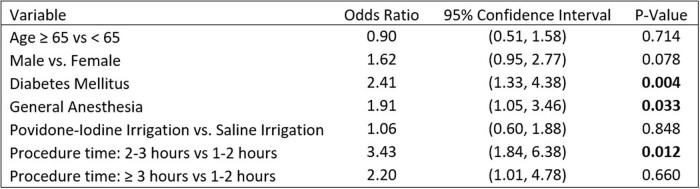
Multivariable analysis adjusted for age, sex, diabetes mellitus, anesthesia type, and procedure time. Showing odds of developing a surgical site infection.

**Table 3.**
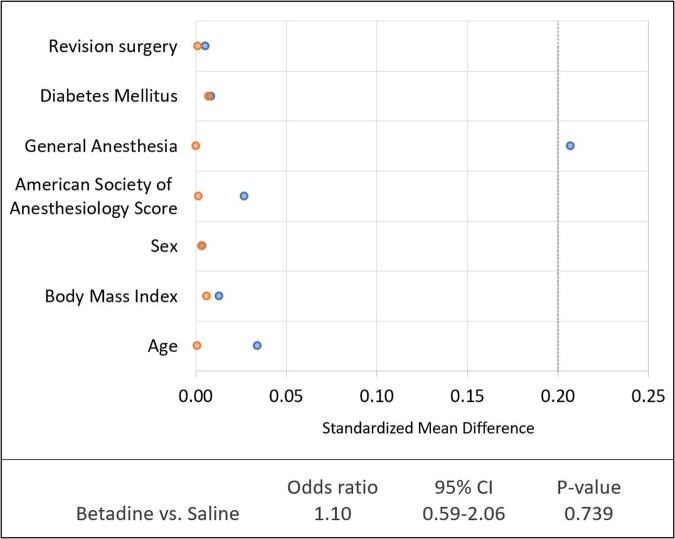
Graph of standardized mean differences before and after matching. Blue dots represent unmatched cohort. Orange dots represent matched cohort. Table shows odds of developing a surgical site infection for the matched cohort.

**Conclusion:**

Our analysis indicates no difference between povidone-iodine and saline irrigation in reducing SSI after THA/TKA. In situations where financial cost may be a factor in determining whether to use saline or povidone-iodine, we conclude that saline irrigation is equally efficacious and therefore a reasonable choice.

**Disclosures:**

**Eric Smith, MD**, Conformis: Advisor/Consultant|Depuy: Advisor/Consultant

